# An early origin of oxygenic photosynthesis delays the Great Oxidation

**DOI:** 10.1098/rstb.2024.0094

**Published:** 2025-08-07

**Authors:** Julia Elizabeth Horne, Colin Goldblatt, Lee Kump

**Affiliations:** ^1^ School of Earth and Ocean Sciences, University of Victoria, Victoria, British Columbia, Canada; ^2^ Department of Geosciences, The Pennsylvania State University, University Park, PA, USA

**Keywords:** Great Oxidation event, Precambrian, biogeochemical model, nitrogen, phosphorus, oxygen

## Abstract

The Great Oxidation Event (GOE) was the most significant chemical revolution in Earth’s history, occurring 2.4 billion years ago. The metabolism that made this transition possible, oxygenic photosynthesis, may have evolved as early as the Eoarchean (3.5 Ga) and certainly by the end-Archean. A long period with low oxygen was facilitated by rapid atmospheric oxidation reactions prior to ozone layer formation, but the mechanisms controlling the length of the delay remain unknown. In this paper, we use EONS (Earth Oxygenation and Natural Systematics), a new biogeochemical model of the Earth system, to evaluate different scenarios for the evolution of two key metabolic pathways—oxygenic photosynthesis and nitrogen fixation, and inorganic phosphorus cycle boundary conditions to constrain determinants of oxygenation timing. We find, counter-intuitively, that an early origin of oxygenic photosynthesis leads to a longer delay before the GOE, and that the earliest‐modelled origins delay the Great Oxidation the longest in absolute terms. The ultimate control over oxygenation delay is phosphorus availability; a strong productivity bottleneck emerges when oxygenic photosynthesis and nitrogen fixation evolve before the accumulation of significant surface phosphorus reservoirs. This bottleneck is perpetuated by strong ocean redox stratification and efficient phosphorus sequestration, which limit primary productivity and hence oxygen accumulation.

This article is part of the discussion meeting issue ‘Chance and purpose in the evolution of biospheres’.

## 1. Introduction

Earth’s modern atmospheric composition is in large part a result of biospheric reworking: life emerged on an anoxic Earth in the Archean eon, and has since remade the primordial atmosphere into its current form [1]. Simple, single-celled prokaryotes (archaea and bacteria) evolved in the Eoarchean, somewhere between 3.8 and 3.5 Ga [2]; the first (anoxygenic) photosynthesizing organisms are believed to have used $H{,}_{2}$ as reductant before moving on to $\mathrm{Fe}{,}^{2+}$ and $H{,}_{2}O$ [3,4]. Photosynthesizers started producing free $O{,}_{2}$ as a waste product in this reaction by at least the Neoarchean (2.8–2.5 Ga), as evidenced by Fe-Mo isotope fractionation [5] and stromatolite and tufted mat formations, which have been interpreted as remnants of cyanobacterial colonies [6,7]. Molecular clock estimates for the emergence of photosystem II ($H{,}_{2}O$-splitting) suggest this capability arose as far back as the Eoarchean (>3.5 Ga) [8] and oxygen-producing enzymes became widespread by the early Mesoarchean (3.1 Ga) [9]. These age estimates support interpretations of early Archean manganese oxide-enriched sediments as potential evidence for biospheric oxygen production [10]. Gradual accumulation of organic carbon on the emergent continents [11–13], progressive loss of $H{,}^{+}$ to space [14,15] and changes in the flux of reductants to the surface [16–18] drove a decline in reducing capacity that biospheric oxygen production eventually overtook, resulting in the rapid rise in oxygen at the start of the Palaeoproterozoic (2.4 Ga) [19,20]. Why exactly there was a delay between the emergence of oxygen production and the oxygenation of the atmosphere has long been a subject of interest, with theories ranging between physiological limitations and inefficiencies in oxygen producers [21], changing dynamics of primary producers under increasing oxygenation [22,23], geochemical and tectonic shifts in reductant fluxes [16–18], accumulation of organic [11] or phosphorus-rich carbonate [24] sediments on stable continental bodies, atmospheric dynamics [15,25], enhanced phosphorus sequestration under changing ocean chemistry [26,27] and nutrient system feedbacks [28], among others.

The biosphere exists in close communion with the geosphere, and the two systems evolve in concert. Nitrogen and phosphorus, which cycle on geological timescales between the crustal reservoirs and the atmosphere–ocean, are critical components of organic matter and therefore limit where life can flourish and how much is sustainable by the environment. In the modern biosphere, nitrogen is fixed to $\mathrm{NH}{,}_{3}$ by primary producers from unusable but plentiful $N{,}_{2}$; isotopic evidence of nitrogen fixation dates back to 3.2 Ga [29], though it is possible that this biofunctionality developed in bacteria as far back as 3.77 Ga [30]; it remains unclear if $N{,}_{2}$ fixation evolved as a result of evolutionary pressures or pure chance [31]. Phosphorus cycling is more restricted by the geosphere and primarily made available to organisms via continental weathering [32], and as such it forms a critical link between the rock cycle and the biosphere [33]. Other sources of inorganic phosphorus include anoxic seafloor weathering [34] and hydrothermal plumes [35]. Once phosphorus is dissolved in the ocean, its accessibility to the biosphere can be further limited by absorbtion of ferric iron oxides [36,37], formation of vivianite [38], incorporation into aluminium phosphate-sulfate minerals [39,40] or mineralization as fluorapatite with carbonate minerals [32]. Because of limited continental emergence and enhanced phosphorus sinks in an anoxic ocean [27,36,41–43], phosphorus availability may have been substantially restricted during the Precambrian after oxygenic photosynthesis evolved. This phosphate-limited Archean paradigm is supported by several biogeochemical models of the Precambrian [44–48]. Some authors argue that phosphate levels were higher than or near modern values in the late Archean based on shallow water carbonate-hosted phosphate minerals [49–51] and apatite particles in deep ocean banded iron formations [52], requiring productivity be limited by other species [53,54].

In this work, we use the Earth Oxygenation and Natural Systematics (EONS) model [28] to test different scenarios for the Great Oxidation Event (GOE), focusing on biospheric controls on primary productivity and oxygenation. This fully coupled biogeochemical model of the C-N-O-P cycles spanning the atmosphere–ocean and geosphere uses relatively few, simple external forcings (including linearly increasing solar constant and linearly declining mantle reductant outflux) to evolve the Earth system from the start of the Eoarchean to the present. These forcings include the timing of key biosphere transitions such as the emergence of oxygenic photosynthesis and nitrogen fixation, enabling us to compare different scenarios with early and late emergence for either metabolism. In order to assess the relative control of the phosphorus cycle on oxygenation, we modify an initial condition that dictates the rate of growth for the continental phosphorus reservoir.

We begin our discussion of Earth’s oxygenation in §2, with a breakdown of how oxidizing and reducing power shifts across this transition, and how that shift relates to the biosphere. In §3 we consider the effects of different start times for oxygenic photosynthesis and nitrogen fixation on the GOE, and major differences between early and late scenarios. We perform a simple sensitivity test for continental phosphorus growth and its effect on GOE timing in §4 to clarify the ultimate controls on oxygenation timing.

## 2. Bistability and atmospheric oxygenation

Before one can understand the Great Oxidation Event as a complex intersection of long-term biological and geological influences, one must clarify the mechanisms by which atmospheric oxygenation proceeds during transition. Over geologic timescales, oxygenation occurs in stepwise fashion, with a rapid, nonlinear leap between stable states characterizing the brief event known as the GOE. Goldblatt *et al.* [25,55] described how low to moderate biological productivity allows for the persistence of a stable, low-oxygen atmosphere for hundreds of millions of years following the beginning of oxygenic photosynthesis. Two distinct stable states arise because when oxygen levels are low (p${\text{O}}_{2}<10^{-6}$ bar) its mutual destruction with methane limits its atmospheric lifetime, whereas at higher oxygen levels (p${\text{O}}_{2}\geq 10^{-3}$ bar) an ozone layer begins to form, shielding tropospheric ${\text{H}}_{2}\text{O}$ from high-energy UV radiation and reducing ${\text{OH}}^{-}$ production and ${\text{CH}}_{4}$ oxidation, thereby extending oxygen’s photochemical lifetime [20,25]. The transition between these states is a function of net oxygen production and the reducing capacity at the Earth’s surface.

### (a) A framework for oxygen level

Goldblatt *et al.* [25] made a model for the GOE comprising oxygen, methane and organic carbon fluxes between the atmosphere–ocean and the continents, illustrating how the transition from anoxic to oxic states occurs by solving their model for ${\text{O}}_{2}$ steady state: Goldblatt *et al.* [55] shows that oxygen level is a function of productivity ($N_{pp}$), reductant influx (r) and the change in the geologic organic carbon reservoir (ΔC), such that the Great Oxidation occurs at a threshold value.

We use this framework to illustrate how the GOE proceeds in EONS. Methane oxidation is updated from this original treatment [28], such that solving for this balance results in a slightly different equation (see appendix A), using similar terminology:


(2.1) $${\text{[O]}}_{2}=f\left(\frac{N_{pp}}{(r-\Delta C{)}^{\frac{1}{2}}}\right).$$

**Figure 1.**
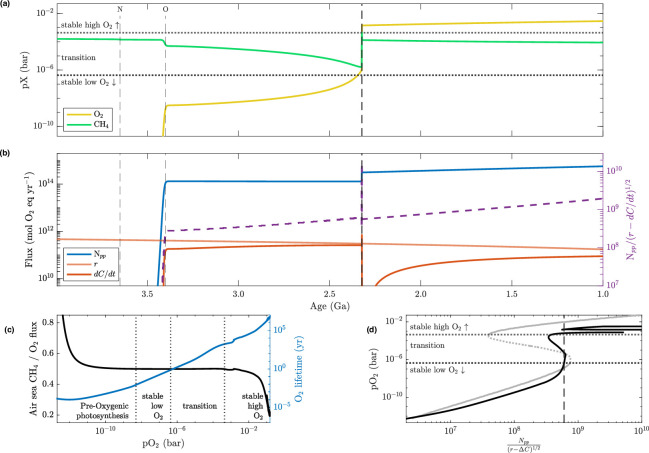
Bistability controls on oxygenation. The GOE occurs as net primary productivity ($N_{pp}$) is significantly greater than the square root of difference between reductant influx (r) and net geologic organic carbon accumulation (ΔC), based on a relationship described by Goldblatt *et al.* [25] (a) Atmospheric mixing ratios for oxygen and methane throughout the Precambrian; (b) left *y*-axis shows components of the oxygen balance equation as fluxes through time in mol $O{,}_{2}$ equivalents/yr (denoted in legend), while right *y*-axis shows the ratio between those fluxes as a dashed line (see equation (2.1)); (c) the ratio of $\mathrm{CH}{,}_{4}$ to $O{,}_{2}$ flux to the atmosphere is shown on the left axis, and the lifetime (yr) of atmospheric oxygen is shown on the right axis, both as functions of atmospheric oxygen level (bar); (d) oxygen level as a function of the oxygen balance equation between net production over net reduction. The grey line in (d) shows a theoretical curved based on fixed r and ΔC values against a range of $N_{pp}$ (the unstable range is shown as a dotted line), while the black curve is calculated from model output of $N_{pp}$, r and ΔC fluxes. Thresholds for stable anoxic (p$O{,}_{2}<10^{-6}$ bar) and oxic (p$O{,}_{2}\geq 10^{-3}$ bar) atmospheric and the unstable transition conditions are noted in panels (a) and (d) by horizontal dotted lines. Vertical dotted lines in (c) denote oxygen levels characteristic of pre-oxygenic photosynthesis (before 3.5 Ga), low $O{,}_{2}$, GOE transition and high $O{,}_{2}$ atmospheric conditions. Thin vertical dashed lines in (a) and (b), labelled *N* and *O*, denote the start of nitrogen fixation and oxygenic photosynthesis, respectively, in this nominal model run. Thick vertical dashed lines in (a), (b) and (d) denote the time or ratio when the ozone layer begins to form, corresponding to the atmosphere's transition from stable anoxic conditions into the nonlinear Great Oxidation; this transition period is characterized by an increase in atmospheric $O{,}_{2}$ lifetime by three orders of magnitude.

All of these fluxes are calculated in units of mol ${\text{O}}_{2}$ equivalents yr^–1^, and we illustrate how their relationship dictates the changing balance of oxygen and methane in the atmosphere through time in a standard run of EONS in figure 1. In this scenario, nitrogen fixation begins at 3.8 Ga, followed by oxygenic photosynthesis at 3.5 Ga. We impose a gradual expansion of these metabolisms over 100 million years, using a logistic function to smoothly activate a forcing from zero to 1, such that the timed onset of these productivity pathways does not create untenable numerical instabilities in the model (see [28] for more detail on the implementation of biological transitions in EONS).

### (b) Oxygenation controls

In the simplest terms, the GOE occurs once oxygen accumulates sufficiently for an ozone layer to form. There are two fundamental balances which result in equation (2.1); first that the production of methane and oxygen by the biosphere balances methane oxidation in the atmosphere,


(2.2) $${\text{CO}}_{2}+2\;{\text{H}}_{2}\text{O}\overset{\text{biospheric production}}{\underset{\text{atmospheric destruction}}{⇌}}{\text{CH}}_{4}+2{\text{O}}_{2}$$

second that the net geological source of reductant (as methane) balances hydrogen escape. A higher net reductant flux (r−ΔC) gives higher methane and lower oxygen, whereas a higher net primary productivity ($N_{pp}$) gives higher oxygen. These are combined via equation (2.1) to show that a simple threshold is sufficient to describe when the GOE occurs, when $N_{pp}/(r-\Delta C{)}^{1/2}$$>6\times 10^{8}$ (figure 1*d*). Unlike the model of Goldblatt *et al.* [25], EONS includes dynamic fluxes which respond to environmental conditions; this means that $N_{pp}$, r and ΔC are not fixed values but rather functions of interacting systems. We define the relationship of these terms to fluxes in EONS in appendix A. Stability of two distinct oxygen levels is a robust feature of the GOE. Whether these oxygen levels are ‘bistable’ (i.e. overlapping) is model dependent, and determined by the network of oxygen feedbacks considered [15,22,56]: in EONS, the addition of oxygen-sensitive biosphere and weathering feedbacks makes the produced GOE a sharp, nonlinear transition in oxygen state, rather than exhibiting true bistability as in Goldblatt *et al.* [25] because the simplified assumption of biosphere production of methane and oxygen in a 0.5 ratio breaks down across the transition (figure 1c,d).

The Great Oxidation Event produced by EONS is necessarily a complex one. Following the establishment of oxygenic photosynthesis, $N_{pp}$ stabilizes at a level much greater than the flux of reducing species to the surface (figure 1b, 3.5 Ga to 2.5 Ga). Yet this outpacing of net surface reductant flux is insufficient to oxygenate the atmosphere at 3.5 Ga, because the level of Archean p${\text{CH}}_{4}$ is still high enough to suppress oxygen’s atmospheric lifetime (figure 1a,c). Because the biosphere produces ${\text{CH}}_{4}$ and ${\text{O}}_{2}$ in a near balanced 0.5 ratio through this transition period, and because $N_{pp}$ reaches a stable level, the process of oxygen’s accumulation in the atmosphere is gradual.

The rapid GOE transition, occurring around 2.4 Ga, initiates when mantle reductant flux, r, becomes sufficiently smaller than primary production such that enough oxygen can accumulate to begin the formation of an ozone layer (figure 1a,b). Further oxygenation gradually follows as oxygen’s atmospheric lifetime lengthens with this additional shielding (figure 1c). At the threshold of stable oxic conditions (p${\text{O}}_{2}\geq 10^{-3}$ bar), oxidative continental weathering drives a decline in ΔC and increase in $N_{pp}$ because the enhanced flux of continental organic N and P to the ocean boosts primary productivity while also having the immediate effect of consuming atmospheric ${\text{O}}_{2}$ in the weathering reaction (figure 1a,b, at 2.4 Ga). Oxygen’s rise is sharply curtailed as it reaches $10^{-3}$ bar by the activation of oxidative continental weathering (causing a significant drop in ΔC) and enhanced methanogenesis by the more productive biosphere. After the ocean's organic carbon reservoir is sequestered several million years later, oxygen and methane achieve a new atmospheric balance that persists until the start of the Neoproterozoic (1 Ga; figure 1a).

Thus, the behaviour of both $N_{pp}$ and r−ΔC changes dramatically across the GOE in feedbacks that obfuscate the ultimate controls on oxygenation. Horne & Goldblatt [28] demonstrated the sensitivity of the GOE to changing parameterizations of r (i.e. $F_{\text{mantle,FeO}}$), which declines through time. In the following sections, we demonstrate that nitrogen and phosphorus availability controls the growth of $N_{pp}$ and thus the timing of the GOE.

## 3. The emergence of oxygen and nitrogen production

The road to the GOE was paved by marine primary producers. Beginning sometime in the Archean [4–6,8–10], oxygenic photosynthesis became an efficient pathway that revolutionized the productive capacity of the biosphere, enabling it to rapidly expand beyond its previous electron donor-limited regime [57]. Free ${\text{O}}_{2}$ is only produced when primary production is greater than aerobic remineralization, which uses available ${\text{O}}_{2}$ to reprocess organic matter into its constituent nutrients.

Oxygenation begins in the shallow photic zone of the ocean where primary production occurs, whereas dissolved oxygen is substantially consumed by remineralizing bacteria as sinking (exported) organic matter reaches the deeper ocean. Vertical zoning of production and processing promotes a strong redox gradient in the Precambrian ocean between an oxic surface and anoxic deep. This redox stratification scheme is characteristic of the ocean until the late Neoproterozoic or early Phanerozoic [58,59], when the deep ocean finally oxygenated.

Primary producers are dependent on their environmental conditions, particularly the nutrients at their disposal. A critical step in the expansion of the biosphere was the generation of a reliable supply of fixed nitrogen. There are minor abiotic sources of fixed nitrogen in EONS (volcanic, metamorphic and reduced mantle outgassing) that amount to approximately $10^{11}$ mol N yr^−1^, which is two orders of magnitude lower than fixed nitrogen production by the modern biosphere. Since the Archean [29,30], organisms have fixed ammonia (${\text{NH}}_{3}$) from atmospheric ${\text{N}}_{2}$ under conditions where its dissolved concentration limited organic matter production [60]. When this functionality emerges relative to oxygenic photosynthesis determines whether or not fixed nitrogen is a limitation on $N_{pp}$ in the Archean; essentially, the timing of this evolutionary leap is the boundary condition that dictates how strongly the N cycle controlled oxygenation.

**Figure 2.**
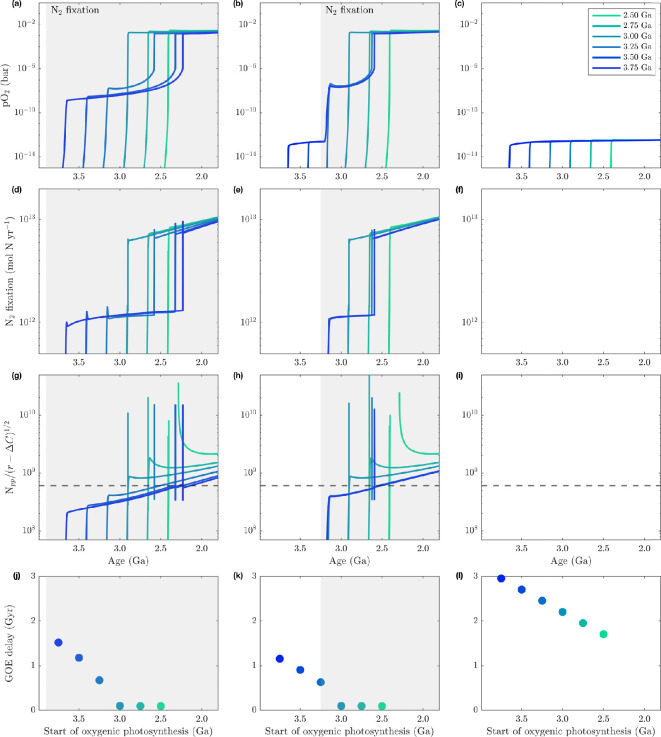
Metabolic controls on oxygenation. We illustrate the relevance of nitrogen fixation evolution, on the GOE in three cases. From left to right: first column (a,d,g,j) shows scenarios for Eoarchean fixation; middle column (b,e,h,k) shows scenarios for Palaeoarchean fixation; right column (c,f,i,l) shows scenarios without fixation. In each panel, the onset of oxygenic photosynthesis is denoted by sequential line (or dot) colour, ranging from 3.75 Ga (dark colors) to 2.5 Ga (light colour), as summarized in the legend in (c), which is for all panels; the first three rows share the same *x*—axis, model time in Ga—whereas the bottom row panels have their own *x*-axis. The period of active biological $N{,}_{2}$ fixation is denoted by the grey shaded field in each panel; first row (a–c) shows oxygen partial pressure, second row (d–f) shows nitrogen fixation flux in mol N yr^−1^, third row (g–i) shows the oxygen balance equation of net oxygen production and net reductant influx, with the horizontal dashed line denoting the threshold for triggering ozone layer formation and the Great Oxidation Event; fourth row (j–l) shows the delay between onset of oxygenic photosynthesis and the oxygenation of the atmosphere (when p$O{,}_{2}>10^{-6}$ bar) for each scenario as a function of the age of oxygenic photosynthesis (in Ga).

To illustrate this control on the GOE, we impose different start times for the evolution of oxygenic photosynthesis relative to nitrogen fixation as boundary forcings in EONS; the resulting oxygenation scenarios are shown in figure 2, including an extreme case where $N{,}_{2}$-fixation never evolves (third column: *c*, *f*, *i* and *l*).

Atmospheric oxygenation occurs in every scenario in which the difference between oxygen production and net reducing capacity exceeds the critical threshold of $6\times 10^{8}$ (figure 2a–f), which only occurs if nitrogen fixation is active. Without an ${\text{N}}_{2}$ fixation pathway, oxygenation is impossible until the Neoproterozoic colonization of land by fungi (750 Ma); strong limitation of $N_{pp}$ by nitrogen ensures the ratio between oxidizing and reducing fluxes is orders of magnitude below the critical threshold for triggering the GOE, and the Earth remains anoxic throughout the Proterozoic (figure 2i). Generally, oxygen evolves in stepwise fashion, with larger increases associated with nitrogen-fixing biospheres (figure 2a–f). Atmospheric oxygenation is capped at a lower threshold in the period before $N{,}_{2}$-fixation starts ($<10^{-12}$ bar; figure 2b,c,e,f); in these scenarios, primary productivity is restricted by low fixed N, which suppresses oxygen’s rise until after fixation evolves (figure 2g–i). After its emergence, nitrogen fixation rapidly increases to match demand on fixed N, and yet stalls well below modern levels prior to oxygenation ($1\times 10^{13}$ mol N yr^−1^; figure 2d,e). Following the GOE, fixation rises to near modern rates, indicating a significant increase in demand for fixed nitrogen after this transition.

### (a) The paradox of primordial metabolisms

This exercise presents a somewhat counter-intuitive result: starting oxygen production earlier does not oxygenate the atmosphere faster. Scenarios with earlier oxygenic photosynthesis experience longer delays before atmospheric oxygenation (figure 2a,b; darker lines, evolving before 3 Ga). Meanwhile, scenarios with younger oxygenic photosynthesis (figure 2a,b; lighter lines, after 3 Ga) experience near instantaneous GOEs. The most ancient (Eoarchean) oxygenic photosynthesis and ${\text{N}}_{2}$-fixation scenarios actually yield the longest delays between the start of oxygen production and the Palaeoproterozoic GOE (dark lines/dots in figure 2a,j); this implies that alleviating N limitation early—thus increasing $N_{pp}$—makes oxygenation *more difficult* rather than easier.

The alleviation of one biospheric limitation begets another, stronger limitation; early, vigorous biospheric production must initiate a feedback that prolongs oxygen’s rise. Atmospheric oxygenation is fundamentally a product of primary productivity level (§2b); not only does production of oxygen need to outpace reductant influxes in order to initiate the GOE, but it must outpace them by many orders of magnitude. Not long after oxygenic photosynthesis evolves, oxygen production eclipses reductant fluxes by two orders of magnitude (figure 1b). Yet we find that even extended periods of elevated $N_{pp}$ will not result in early oxygenation if productivity stabilizes at a level below the critical threshold (figure 2a,b,g,h). Higher Archean $N_{pp}$ is correlated with an extended period of slow p${\text{O}}_{2}$ growth from $10^{-9}$ to $10^{-6}$ bar (figure 2a), which does not occur if nitrogen fixation evolves after oxygenic photosynthesis and p${\text{O}}_{2}$ remains below $10^{-9}$ bar (figure 2b, dark lines). Curiously, the GOE begins to take on uniform behaviour if oxygenic photosynthesis evolves between 3 and 3.25 Ga, regardless of the order in which these metabolisms evolve.

This stagnation reveals a systemic p${\text{O}}_{2}$ sensitivity that fundamentally shifts when fixed N becomes available, but only up to a certain point in absolute time. Importantly, the pre-GOE plateau in nitrogen fixation rate implies that other limitations on the biosphere take hold and contribute to this delay (figure 2d,e). Following nitrogen fixation evolution, the ultimate limitation on the biosphere becomes the availability of phosphorus, which is controlled foremost by the emergence of continents.

## 4. Oxygenation delay and phosphorus

In the modern marine biosphere, dissolved phosphorus is in high demand. This contrasts particularly with nitrogen, which is readily attainable thanks to the work of ${\text{N}}^{2}$-fixers, whereas phosphorus is ensnared in a longer cycle of geologic processing, having no atmospheric reservoir like ${\text{N}}^{2}$. In a post‑N-limited world, phosphorus cycling dictates the growth of primary productivity and its dwindling supply generates a biospheric bottleneck, the strength of which determines length of delay between the evolution of oxygenic photosynthesis and the Great Oxidation Event.

**Figure 3.**
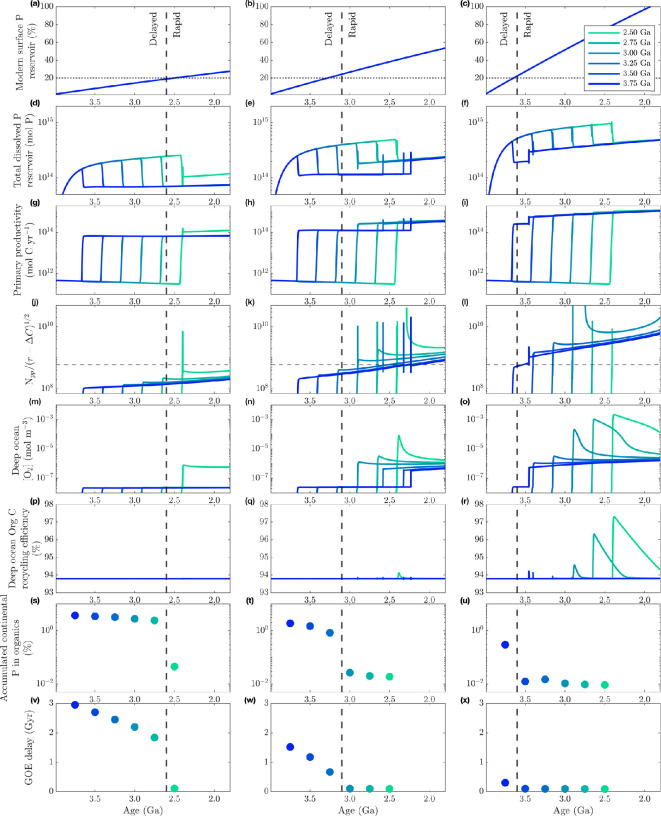
Effect of P availability on GOE delay. We impose different surface P reservoir growth rates: from left to right, the left column (a,d,g,j,m,p,s,v) is half the nominal rate, middle column (b,e,h,k,n,q,t,w) is nominal and right column (c,f,i,l,o,r,u,x) is double the nominal rate. Dashed vertical lines denote the ‘tipping point’ in oxygenation delay, with billion-year delays occurring in instances to the left of the line, and negligible delays to the right. First row (a–c) shows total surface P (all continental, ocean and sedimentary reservoirs) as a percentage of the modern; second row (*d*–*f*) shows total ocean $H{,}_{3}\mathrm{PO}{,}_{4}$ (mol P); third row (*g*–*i*) shows primary productivity (mol C yr^−1^); fourth row (j–l) shows the balance of net oxidation and net reductant flux$N_{pp}/(r-\Delta C{)}^{1/2}$ , with the horizontal dashed line denoting the threshold for triggering atmospheric oxygenation; fifth row (m–o) shows deep ocean $\left[O{,}_{2}\right]$ (mol m${,}^{-3}$); sixth row (p–r) shows the percentage of exported organic matter that is remineralized in the deep ocean; seventh row (s–u) shows the percent of total continental phosphorus accumulated as organic P prior to the GOE; eighth row (v–x) shows the delay between onset of oxygenic photosynthesis and the oxygenation of the atmosphere (when p$O{,}_{2}>10^{-6}$ bar) for each scenario. In each panel, the onset of oxygenic photosynthesis is denoted by sequential line (or dot) colour, ranging from 3.75 Ga (dark colours) to 2.5 Ga (light colours); in the first row these lines overlap, as surface P growth scenarios are the same across each test case for that column. The horizontal dotted lines in (a)–(c) denote approximately 20% of the modern surface P reservoir, a critical threshold for rapid oxygenation. The onset of $N{,}_{2}$ fixation is the same for all cases, starting at 3.8 Ga. Note: the last two rows of scatter plots show the age of initial oxygenic photosynthesis on the *x*-axis.

We demonstrate this control by employing a simple sensitivity test on P surface influx, shown in figure 3, in which we vary only the rate at which phosphorus is transferred from the mantle to the continents; the nominal treatment for surface phosphorus reservoir growth is shown in the middle column (b, e, h, k, n, q, t, w); slower and faster rates of growth are shown in the left (a, d, g, j, m, p, s, v) and right (c, f, i, l, o, r, u, x) columns, respectively. The final row of figure 3(v–x) shows the delay between the start time for oxygenic photosynthesis and the oxygenation of the atmosphere, with the tipping point between long and negligible delays denoted by vertical dashed line.

Continental weathering supplies the ocean with dissolved phosphorus; in EONS, the rate of continental weathering for any mineral species is primarily a function of the size of its continental reservoir, which grows from sedimentary accretion, burial of sediments on continental shelves and mantle eruptions [28]. Some estimates for the Precambrian crustal inventory of igneous phosphorus indicate rapid growth to near-modern P concentrations by the mid-Archean [61,62], though the abundance and composition of continental crust are also significant controls on phosphorus flux to the ocean [63]. Our surface P reservoir here is the amount of phosphorus, relative to modern, that is capable of being transported to the ocean. This model does not include other sources of phosphorus, such as seafloor weathering [34] or hydrothermal input [35,52], which may have allowed for higher dissolved phosphorus concentrations on an early Earth with even less continental emergence. EONS initiates with mineral species primarily contained within the mantle, assuming small continents at the start of the Eoarchean. During the first few billion years of the model run, mantle eruptive emplacement is the dominant pathway by which continental phosphorus grows. We are able to test different scenarios of surface P growth rate by simply varying the size of this initial mantle reservoir.

### (a) Phosphorus availability dictates GOE timing

Figure 3 rows 1–4 (a–l) illustrates how phosphorus availability controls the timing of the GOE after oxygenic photosynthesis begins. The amount of inorganic phosphorus in continents supports the dissolved phosphorus reservoir in the ocean through weathering; faster growth of the continental P reservoir allows for higher dissolved P when oxygenesis begins (figure 3a–f). At that point, the biosphere goes from being electron donor-limited to phosphorus-limited. The size of the dissolved phosphorus reservoir therefore dictates primary productivity ($N_{pp}$; figure 3g–i). Lower dissolved phosphorus keeps $N_{pp}$ below the critical threshold for longer (figure 3d,j), whereas higher dissolved phosphorus means $N_{pp}$ can exceed the critical threshold when oxygenic photosynthesis begins (figure 3f,l). This results in a long GOE delay in the former case, and an immediate GOE in the latter.

The size of the surface P reservoir when oxygenic photosynthesis evolves is thus inversely correlated with the length of delay before the Great Oxidation; the biosphere becomes capable of near instant oxygenation if there is >20% modern P at the surface (figure 3a–c,v–x); in the nominal case, this threshold is achieved around 3.25 Ga, which explains the time-dependency noted earlier (§3a).

### (b) Ocean redox stratification drives phosphorus sequestration

The development of a more productive biosphere and increasing surface ${\text{O}}_{2}$ imposes a negative feedback on biospheric expansion by sequestering phosphorus. Figure 3 rows 5–7 (m*–*u) illustrates why this feedback occurs, and how it is strengthened in cases with more ancient oxygenesis.

The phosphorus bottleneck ultimately results from ocean redox stratification. In this pre-GOE regime, more organic matter is produced and exported from the surface ocean to a deep ocean that is not significantly oxygenated. Oxygen produced in shallow waters enables localized aerobic remineralization, which in turn perpetuates high photosynthetic primary production by resupplying nutrients. Because primary production is absent below the photic zone, whatever oxygen is able to mix downward is consumed in organic matter remineralization, thus perpetuating strong anoxia in the deep ocean that is only somewhat alleviated after the GOE (figure 3m–o).

Following the evolution of oxygenic photosynthesis, orders of magnitude more organic material is exported to the deep ocean (figure 3g–i), yet the same *proportion* of that material escapes remineralization. Anaerobic remineralization, which dominates in the anoxic deep ocean, is less efficient than remineralization using oxygen. Prior to full ocean oxygenation in the Phanerozoic, only around 94% of organic matter sinking into the deep ocean is remineralized in EONS (figure 3p–r); notably, the efficiency of remineralization in the deep ocean is equivalent in the pre- and post-oxygenesis periods for all scenarios, and transient increases are associated only with oxidation events. If there is no penetration of oxygen into the anoxic deep ocean, phosphorus levels (and therefore productivity) are suppressed by the biological pump (figure 3e,n,q, dark lines); ocean phosphorus levels are so consistent through the GOE delay regardless of when oxygen production starts because the difference in oxygen levels between the surface and deep ocean is similarly consistent (figure 3d–f,m–o). Until a transient increase in deep ocean oxygen level allows for more phosphorus recycling (figure 3p–r), the redox-stratified ocean falls into a negative feedback loop that maintains phosphorus levels, and thus productivity, constant. Even a small, short lived increase in deep ocean recycling is sufficient to push the system out of this low-phosphorus state.

Scenarios with more ancient oxygenic photosynthesis (blues) persist for longer in a period of strong organic P sequestration for longer, and have a higher portion of total continental P ‘locked away’ as a result. Figure 3, row 7 (s*–*u) shows the total accumulated reservoir of organic phosphorus in continents prior to the Great Oxidation Event (expressed as a percentage of total continental phosphorus), plotted against the age of oxygenic photosynthesis. Earlier oxygenesis correlates to more organic P sequestration. Since oxidative weathering does not initiate until after the atmosphere becomes oxic (p${\text{O}}_{2}\geq 10^{-3}$ bar), this portion of the surface phosphorus reservoir becomes effectively inaccessible to the ocean biosphere. This stalls the growth of the dissolved P reservoir (figure 3d–f) and delays the increase in $N_{pp}$ necessary to cause the GOE (figure 3g–l). This feedback explains why nitrogen fixation emerging in the Palaeoarchean—after oxygenic photosynthesis—produced shorter GOE delays (§3a): in those scenarios, $N_{pp}$ was strongly nitrogen-limited, which kept p$O{,}_{2}$ low and prevented the development of ocean redox stratification. Such a weak biosphere in a uniformly anoxic ocean allowed dissolved phosphorus to accumulate.

## 5. Conclusions

The Great Oxidation Event, the most significant chemical revolution in Earth’s history, emerged from a confluence of gradual shifts in reducing and oxidizing capacity at the Earth’s surface. The GOE occurs as a nonlinear transition as the ozone layer begins to form, which itself is triggered at a threshold atmospheric oxygen level. This threshold is well predicted in EONS by a ratio of net primary productivity to the square root of the difference between reductant supply and net organic carbon burial [25,28].

Biogeochemical models such as EONS can produce a multitude of scenarios for the GOE after the onset of oxygenic photosynthesis, but all scenarios fall into one of two primary categories: rapid or delayed oxygenation. A critical pattern we find among these scenarios is that more ancient oxygenic photosynthesis is associated with longer delays in GOE onset. This association is strengthened if nitrogen fixation is similarly ancient; we find that an Archean oxygenic biosphere that is not limited by fixed-N availability would result in a multi-billion year delay before the GOE. We conclude that a longer history of biospheric oxygen production is not necessarily conducive to atmospheric oxygenation.

We further demonstrate the critical roles that N and P cycles play in Earth’s oxygenation. Without nitrogen fixation, the Earth does not oxygenate in the Proterozoic because net primary production remains significantly lower than the critical threshold required to overwhelm net reductant influx. However, nitrogen fixation is well constrained to having evolved in the Archean, and according to our results therefore should not have contributed significantly to the GOE delay. Phosphorus availability, which is a function of continental emergence, dictates the length of delay after oxygenesis. This is supported by the correlation between the growth of the continental igneous phosphate reservoir and punctuated oxygenation events [61]. Surface P reservoir in excess of 20% of modern levels is required to sustain sufficient net primary production to outpace Palaeoproterozoic reducing capacity and trigger oxygenation. Phosphorus availability in the pre-GOE ocean falls into a critical negative feedback with primary production because the Precambrian redox-stratified ocean promotes organic-bound P sequestration, which cannot be returned to the ocean until oxidative weathering begins post-GOE. The omission of additional sources of phosphorus in EONS, such as seafloor weathering and hydrothermal vents, provides additional areas of inquiry for better characterizing the nature of phosphorus cycle control on the GOE delay. Our results suggest that the total amount of phosphorus at the surface constitutes the ultimate control on the delay in oxygenation, but the amount of that surface P reservoir attributable to continental sources will likely decline from the 20% found here if these other sources are included. Therefore, Earth system models assuming limited sources of dissolved phosphorus will produce longer GOE delays, particularly when combined with the assumption of early biospheric oxygen production.

A biogeochemical model like EONS, which consists of intricately coupled C-N-O-P systems spanning the atmosphere, ocean, biosphere and geosphere, provides nuanced insight into critical transitions such as the Great Oxidation Event. There are many theories regarding Earth’s oxygenation pathway, very few of which are mutually exclusive. Models that suggest geologic or tectonic controls are often models of geologic systems, biological changes are hinted at by metabolic and biosphere evolution models, and atmospheric chemistry drivers are implicated by models of atmospheric reactions. That EONS comprises many systems, reactions and spheres makes it a step forward in resolving the many changes across the Earth system that coincided with, and contributed to, the Great Oxidation Event. Stepwise oxygenation is both a product of and itself produces feedbacks within biogeochemical cycles, which confound even apparently logical assumptions, such as early oxygen production promoting early atmospheric oxygenation.

## Data Availability

The EONS model v.1.0 is licensed under MIT and available for download on [64].

## Apendix A. Derivation of oxygen balance

We derive the balance of net primary production ($N_{pp}$), mantle reductant flux (r), and net organic carbon accumulation (ΔC) by modifying the equations from Goldblatt *et al.* [55], updated with a new oxygen dependency and exponent for methane photo-oxidation ($\Psi_{{\text{O}}_{2}}$).

The steady-state solutions for oxygen, methane and organic carbon are found when the following are set to equal zero:

(A 1)dCH4dt=12ΩO2(Npp+r)−s[CH4]−12ΨO2([CH4][O2])12−12ΩO2(β(Npp+r)−wC)(A 2)dO2dt=ΩO2Npp−(1−ΩO2)r−s[CH4]−ΨO2([CH4][O2])12+(1−ΩO2)(β(Npp+r)−wC)(A 3)dCdt=β(Npp+r)−wC

where $\Omega_{{\text{O}}_{2}}$ is the fraction of oxygen produced by the biosphere that reaches the atmosphere (i.e. the portion not aerobically remineralized, therefore a function of oxygen), β is the organic carbon burial fraction, s is hydrogen escape rate, w is the rate of oxidative weathering and C is the crustal organic carbon reservoir. For our purposes, finding the balance of methane and oxygen fluxes that produce atmospheric steady state, geologic reservoirs are considered constants as they change over significantly longer timescales.

Therefore, we solve for simplified atmospheric steady state at fixed ΔC; this term replaces time-dependent change, $\frac{dC}{dt}$ equation (A 3). Steady-state methane should occur at a 2 : 1 stoichiometric ratio with oxygen, so we double equation (A 1) and subtract equation (A 2):

(A 4)
$$\begin{array}{rl} 0 & =\left(\Omega_{O_{2}}\left(N_{pp}+r\right)-2s\left[{CH}_{4}\right]-\Psi_{O_{2}}{\left(\left[{CH}_{4}\right]\left[O_{2}\right]\right)}^{\frac{1}{2}}-\Omega_{O_{2}}\Delta C\right)\\ & -\left(\Omega_{O_{2}}N_{pp}-\left(1-\Omega_{O_{2}}\right)r-s\left[{CH}_{4}\right]-\Psi_{O_{2}}{\left(\left[{CH}_{4}\right]\left[O_{2}\right]\right)}^{\frac{1}{2}}+\left(1-\Omega_{O_{2}}\right)\Delta C\right)\\ 0 & =r-\Delta C-s\left[{CH}_{4}\right][{CH}_{4}]=\frac{r-\Delta C}{s}\\ \text{therefore:}\\ \left[{\text{CH}}_{4}\right] & =\frac{r-\Delta C}{s} \end{array}$$

Substituting this solution into equation (A 2) allows us to solve for simplified oxygen steady state given balanced methane, isolating all $\Omega_{{\text{O}}_{2}}$ terms on one side and cancelling:

(A 5)
$$\begin{array}{rl} 0 & =\Omega_{{\text{O}}_{2}}N_{pp}-(1-\Omega_{{\text{O}}_{2}})r-s(\frac{r-\Delta C}{s})-\Psi_{{\text{O}}_{2}}(\frac{r-\Delta C}{s}[{\text{O}}_{2}]{)}^{\frac{1}{2}}+(1-\Omega_{{\text{O}}_{2}})\Delta C\\ s(\frac{r-\Delta C}{s})+\Psi_{{\text{O}}_{2}}(\frac{r-\Delta C}{s}[{\text{O}}_{2}]{)}^{\frac{1}{2}} & =\Omega_{{\text{O}}_{2}}N_{pp}-(1-\Omega_{{\text{O}}_{2}})r+(1-\Omega_{{\text{O}}_{2}})\Delta C\\ r-\Delta C+\Psi_{{\text{O}}_{2}}(\frac{r-\Delta C}{s}[{\text{O}}_{2}]{)}^{\frac{1}{2}} & =\Omega_{{\text{O}}_{2}}N_{pp}-r+\Omega_{{\text{O}}_{2}}r+\Delta C-\Omega_{{\text{O}}_{2}}\Delta C\\ \Psi_{{\text{O}}_{2}}(\frac{r-\Delta C}{s}[{\text{O}}_{2}]{)}^{\frac{1}{2}} & =\Omega_{{\text{O}}_{2}}N_{pp}+\Omega_{{\text{O}}_{2}}r-\Omega_{{\text{O}}_{2}}\Delta C-2r+2\Delta C\\ \Psi_{{\text{O}}_{2}}(\frac{r-\Delta C}{s}[{\text{O}}_{2}]{)}^{\frac{1}{2}} & =\Omega_{{\text{O}}_{2}}N_{pp}+\Omega_{{\text{O}}_{2}}(r-\Delta C)-2(r-\Delta C)\\ \Psi_{{\text{O}}_{2}}(\frac{r-\Delta C}{s}[{\text{O}}_{2}]{)}^{\frac{1}{2}} & =\Omega_{{\text{O}}_{2}}N_{pp}+(\Omega_{{\text{O}}_{2}}-2)(r-\Delta C) \end{array}$$

Once oxygenic photosynthesis evolves, $N_{pp}\gg |\Delta C|$ and $N_{pp}\gg r$; these assumptions allow us to further simplify and isolate the oxygen-dependent variables $\Omega_{{\text{O}}_{2}}$ and $\Psi_{{\text{O}}_{2}}$ on one side:

(A 6)
$$\begin{array}{rl} \Psi_{{\text{O}}_{2}}(\frac{r-\Delta C}{s}[{\text{O}}_{2}]{)}^{\frac{1}{2}} & =\Omega_{{\text{O}}_{2}}N_{pp}\\ \frac{1}{N_{pp}}(\frac{r-\Delta C}{s}{)}^{\frac{1}{2}} & =\frac{\Omega_{{\text{O}}_{2}}}{\Psi_{{\text{O}}_{2}}[{\text{O}}_{2}{]}^{\frac{1}{2}}} \end{array}$$

The inverse of this becomes an expression encompassing all oxygen dependency in atmospheric steady-state balance with net reducing power, increasing with $N_{pp}$:

(A 7)
$${\left(\frac{\left[{\text{O}}_{2}\right]}{s}\right)}^{\frac{1}{2}}\frac{\Psi_{{\text{O}}_{2}}}{\Omega_{{\text{O}}_{2}}}=\frac{N_{pp}}{(r-\Delta C{)}^{\frac{1}{2}}}$$

hence:

(A 8)
$${\text{[O]}}_{2}=f\left(\frac{N_{pp}}{(r-\Delta C{)}^{\frac{1}{2}}}\right)$$

We relate the three broad oxygen balance terms (net primary production, mantle reductant flux and net organic carbon accumulation, respectively) using fluxes from the EONS model. For net primary production:

(A 9)
$$N_{pp}=F_{\text{prod,}{\text{O}}_{2}}$$

which is the generation of oxygen by primary producers in the ocean (in mol ${\text{O}}_{2}$ yr^−1^). Mantle reductant flux:

(A 10)
$$r=\frac{1}{4}F_{\text{mantle,FeO}}$$

where the flux of mantle reductants to the surface, tracked in mol Fe yr^−1^, is converted to mol ${\text{O}}_{2}$ eq yr^−1^ by dividing by 4. The change in geologic organic carbon reservoir (ΔC) is the net change in unreactive sediment and continental organic carbon (OC) reservoirs:

(A 11)
$$\begin{array}{rl} \Delta C= & F_{\text{burial,OC,}n}+F_{\text{burial,OC,}z}\\ & -F_{\text{wthr,oxi}}-F_{\text{meta,OC}}-F_{\text{volc,OC}}-2F_{\text{mantle,CH4}} \end{array}$$

where a factor of 2 modifies the flux of mantle ${\text{CH}}_{4}$ to the surface such that this equation is in units of mol ${\text{O}}_{2}$ eq. yr^−1^. And we use:

(A 12)
$$\begin{array}{rl} \Omega_{{\text{O}}_{2}}= & 0.8\left(1-(\frac{{\text{[O]}}_{2}}{{\text{[O]}}_{2}+k})\right)\\ \Psi_{{\text{O}}_{2}}= & K_{\text{eff}}({\text{[CH]}}_{4}{\text{[O]}}_{2}{)}^{\frac{1}{2}} \end{array}$$

where $\Omega_{{\text{O}}_{2}}$ is a curve fitted to oxygen air–sea gas exchange over biosphere production of ${\text{O}}_{2}$ ($F_{\text{air-sea,O2}}/F_{\text{prod,O2}}$) used to better map EONS output onto the model of Goldblatt *et al.* [55]. In this fit, $\left[{\text{O}}_{2}\right]$ is the atmospheric concentration of oxygen and k is $3.5\times 10^{-4}$, a transition value for oxygen level in the atmosphere. This approximation is used because in the EONS model, the fraction of organic matter remineralized is not a fixed parameter but sensitive to changing sinking rates and oxygen concentrations, among other factors. The function for $\Psi_{{\text{O}}_{2}}$ is the methane oxidation flux from EONS [28] ($F_{\text{methox}}$); the effective rate constant for this equation ($K_{\text{eff}}$) is a product of ${\text{CH}}_{4}$ and ${\text{O}}_{2}$ atmospheric mixing ratios, fitted to model results from Garduño Ruíz *et al.* [65]:

(A 13)
$$K_{\text{eff}}=3\times 10^{20}\cdot {(4\times 10^{3})}^{[{\left(\frac{-[{\text{O}}_{2}]}{{\text{[O]}}_{2}+10^{-4}}\right)}^{\frac{2}{5}}]}$$
